# What Are the Normal Serum Creatine Kinase Values for Skeletal Muscle? A Worldwide Systematic Review

**DOI:** 10.1111/ene.70240

**Published:** 2025-06-13

**Authors:** Katina Aleksovska, Theodoros Kyriakides, Corrado Angelini, Zohar Argov, Kristl G. Claeys, Marianne de Visser, Massimiliano Filosto, Ivanka Jovanovic, Anna Kostera‐Pruszczyk, Maria Julia Molnar, Sabrina Sacconi, Jochen Schaefer, Gabriele Siciliano, Juan J. Vilchez, Antonio Toscano, Benedikt Schoser

**Affiliations:** ^1^ European Academy of Neurology Vienna Austria; ^2^ Department of Basic and Clinical Sciences University of Nicosia Cyprus; ^3^ Department Neurosciences Campus Pietro D'abano, University of Padova Padova Italy; ^4^ Department of Neurology Hadassah Medical Center and Faculty of Medicine, Hebrew University of Jerusalem Israel; ^5^ Department of Neurology University Hospitals Leuven Leuven Belgium; ^6^ Department of Neurosciences Laboratory for Muscle Diseases and Neuropathies, KU Leuven, and Leuven Brain Institute (LBI) Leuven Belgium; ^7^ Department of Neurology, Amsterdam Neuroscience Amsterdam University Medical Centre, Location Academic Medical Centre, University of Amsterdam Amsterdam the Netherlands; ^8^ Department of Clinical and Experimental Sciences University of Brescia Brescia Italy; ^9^ NeMO‐Brescia Clinical Center for Neuromuscular Diseases ERN EURO‐NMD ASST Spedali Civili Brescia Italy; ^10^ ERN EURO‐NMD ASST Spedali Civili Brescia Italy; ^11^ Executive Director of National Organization of Persons With Disabilities of Serbia Belgrade Serbia; ^12^ Department of Neurology Medical University of Warsaw Warsaw Poland; ^13^ Director of Institute of Genomic Medicine and Rare Disorders Semmelweis University Budapest Hungary; ^14^ Peripheral Nervous System and Muscle Department University Côte D'azur, CHU Nice France; ^15^ University Cote D'azur Institute for Research on Cancer and Aging of Nice (IRCAN), Nice France; ^16^ Department of Neurology Uniklinikum C.G. Carus Dresden Germany; ^17^ Department of Clinical and Experimental Medicine University of Pisa Pisa Italy; ^18^ Neuromuscular Research Group IIS La Fe, Valencia. CIBERER. Universitat de Valencia Valencia Spain; ^19^ CIBERER Universitat de Valencia Spain; ^20^ ERN‐NMD Center for Neuromuscular Disorders of Messina, Department of Clinical and Experimental Medicine University of Messina Messina Italy; ^21^ Friedrich‐Baur‐Institute, Department of Neurology LMU Clinic Munich Munich Germany

**Keywords:** creatine kinase, global, hyperCKemia, myopathy, neuromuscular, normal, systematic review

## Abstract

**Background:**

Serum creatine kinase (CK) has been used as a diagnostic marker for neuromuscular disorders since 1959. As manufacturer‐provided normative data indicate, CK levels can be elevated in normal individuals. Recent evidence suggests these data often underestimate true CK values, which are influenced by age, race, gender, and other physiological factors. Therefore, establishing a reliable normal range for CK is crucial before further investigation, particularly in oligo/asymptomatic patients.

**Objective:**

This systematic review aims to determine the normal CK levels across various populations.

**Methods:**

We systematically reviewed observational studies with a cross‐sectional, descriptive design. We included studies focusing on healthy adults (> 18 years) of any race who aimed to establish normal CK values, including at least 100 participants. We utilized the following databases: PubMed, Embase, and Cochrane.

**Results:**

CK values typically exhibit a non‐Gaussian distribution. Black individuals demonstrate significantly higher CK values compared to Caucasians and Asians. The upper reference limits for CK in Caucasian and Asian males range from 227–440 U/L, while for Black males, it is between 520–810 U/L. The upper reference limits for females range from 135–248 U/L and up to 354 U/L, respectively.

**Conclusions:**

Normal CK values might be higher than previously suggested, especially among specific racial groups. Each laboratory should ideally determine its reference values for CK that reflect its local population.

## 1 Introduction

Creatine kinase (CK) has been recognized as an essential marker for muscle disease since 1959 [1]. It is universally utilized in the diagnostic evaluation of neuromuscular disorders; however, high CK levels often appear in oligo‐symptomatic or asymptomatic individuals. Oligosymptomatic individuals may experience nonspecific muscle symptoms, including myalgia, stiffness, fatigue, or muscle cramps. Such symptoms were common in individuals with either normal CK or hyperCKemia in the Tromsø study, the largest epidemiologic study of hyperCKemia in the general population [2]. The prevalence of hyperCKemia in the Tromsø study, which included 12,828 individuals, was 1.3%. Although CK is a marker of primary muscle disease, various non‐myopathic causes can result in oligo/asymptomatic hyperCKemia. These include strenuous exercise, certain medications (especially statins), as well as cardiac, endocrine, and metabolic conditions, trauma, intramuscular injections, and alcohol, among others [3]. Similarly, normal values are influenced by gender, race, and age [2, 4, 5]. Therefore, establishing an appropriate normal range for CK is crucial before deciding to investigate a patient further, especially if they are oligo/asymptomatic. This decision is especially relevant today, as next‐generation sequencing can offer a prompt and precise diagnosis [6, 7, 8]. The accepted approach to managing muscle disease that presents with oligo/asymptomatic hyperCKemia is to diagnose and treat it early and, when appropriate, provide genetic counseling for the family. The significance of establishing normal CK levels was emphasized by the EFNS Guideline group task force, which discussed the diagnostic approach to oligo/asymptomatic hyperCKemia [3]. We conducted a systematic review to better estimate the normal values of total CK in different racial groups. In the study, the total CK is referred to as CK.

## 2 Methods

To ensure transparency, the protocol employed in this systematic review is published in PROSPERO (https://www.crd.york.ac.uk/prospero/), an international platform for protocols of systematic reviews.

### 2.1 Literature Search Strategy

We included observational studies with a cross‐sectional, descriptive design, including healthy adult populations (> 18 years old) of any race, that aimed to determine the normal values of CK. The eligible studies had to include at least 100 persons. A normal population was defined according to the individual studies' criteria.

We excluded studies in which the sex and race of the individuals under investigation were not reported, as well as studies that focused on myocardial CK, cardiac disease, or other chronic disorders affecting CK levels. Furthermore, studies involving athletes, the effects of physical activity, unhealthy participants, and any other unclassified research on CK levels were also excluded. Finally, we dismissed case studies, case series, conference abstracts, opinion papers, and informal reviews. We aimed to include high‐quality systematic reviews based on the aforementioned inclusion and exclusion criteria. In this instance, the search would have been updated from the last reported date.

We included studies published in English from inception until the last search date (12 December 2022) using PubMed, Embase, and Cochrane. Before finalizing the analyses, we conducted a new search for recent publications (up to 20 May 2024). We searched the references of the eligible studies for additional evidence not retrieved from the databases. Two reviewers searched independently, and all inconsistencies were discussed with a third reviewer. The whole search strategy is described in detail in Appendix 1 and Supporting Information (Appendix 1 includes literature search terms; Tables S1–S3; Figures S1 and S2).

### 2.2 Data Collection and Risk of Bias Assessment

One author collected the data and assessed the risk of bias, and another double‐checked it. If any concerns were raised, they were discussed by the two authors and, if necessary, with a third reviewer.

The following study characteristics were collected in a predefined Excel spreadsheet: first author, year of publication, country of publication, race of the included participants, and the number of participants belonging to each racial group; mean age and standard deviation (SD) of the population; percentage of female participants; the number of individuals according to various age groups (ages 18–40, 41–60, 61–80, > 80); mean, SD, or median CK levels measured in U/L (if any study used different units, these were converted) for each racial group; and the low and high‐end reference values, specifying the method used to calculate them. We also extracted the exclusion criteria and noted whether physical activity or other factors were considered. For the evidence synthesis, we extracted the data and categorized it according to the participants' race/ethnicity as reported in the studies. Based on race/ethnicity and gender, we divided the data into the following categories: Caucasian, Black, Asian, Hispanic, Turkish, and Arab.

The risk of bias was evaluated using the JBI checklist for prevalence studies (https://jbi.global/critical‐appraisal‐tools), as it is the most preferred tool for descriptive cross‐sectional study designs. The checklist comprises nine criteria to assess methodological quality and is primarily designed for studies investigating the prevalence of specific conditions of interest.

### 2.3 Data Analysis

Whenever different units were reported, we converted them to units per enzyme activity per liter (U/L), calculated using an online converter (https://unitslab.com/).

For the statistical analysis, we used the metamedian package in R Studio [9]. We aimed to compare the reference values between various racial groups and estimate the pooled reference values for CK for each racial group separately or within groups that show similar reference values.

Given that normal or log‐normal distribution data were available, we planned to use random‐effects models to calculate means and confidence intervals (95%) of the CK reference values across studies and derive the normative data under the log‐normal distribution assumption. However, the data available from the studies exhibited a non‐normal distribution, and in most cases, the log transformation was inadequate, or it was not reported whether it had normalized the data. Consequently, most studies reported the median values along with the 2.5 and 97.5 percentiles of the population after adjusting for outliers. Recently developed statistical methods aimed at summarizing data that report medians and percentiles were derived from similar approaches suggested in the updated version of the Cochrane Handbook for meta‐analysis of studies that report median [10].

We conducted these analyses to compare reference values across different races and estimate the pooled median values of CK in various racial groups. For these analyses, we included all eligible studies that reported: 1. the first, second (median), and third quartiles; or 2. the mean and standard deviations for the CK values; and 3. the total number of participants. Given that our data were highly skewed, we estimated the CK median for each group using the Quantile Estimation (QE) approach for medians in studies with small sample sizes and the Confidence Distribution (CD) approach when studies had larger sample sizes. We employed the QE for medians approach to compare the CK values among different racial groups. We assessed both within‐ and between‐study heterogeneity using the standard “eyeball test,” the *χ* ^2^ test of heterogeneity, and the *I* ^2^ statistic. When substantial heterogeneity was detected (*p* < 0.05 and *I* ^2^ > 70%), we conducted subgroup analyses based on 1. racial groups, 2. methodological quality, or 3. the exclusion of studies with differing point estimates and significant variations in reference intervals (RI).

The statistical methods described focus solely on estimating medians and have not been developed to assess the pooled lower and upper boundaries of reference values across population groups. As a result, the lower and upper boundaries of the median confidence intervals did not align with the true reference values reported in the individual studies. Although methods for estimating reference ranges are available [11], these require studies to report their mean values or individual participant data, which did not apply to our studies. Given that reference values are crucial for clinical practice, we presented them descriptively, summarizing the ranges reported in the studies.

Finally, alongside the descriptive table, we illustrated our results graphically with forest plots whenever a meta‐analysis was feasible and pertinent (based on the heterogeneity analysis).

## 3 Results

### 3.1 Literature Search and Assessment for Eligibility

**FIGURE 1:**
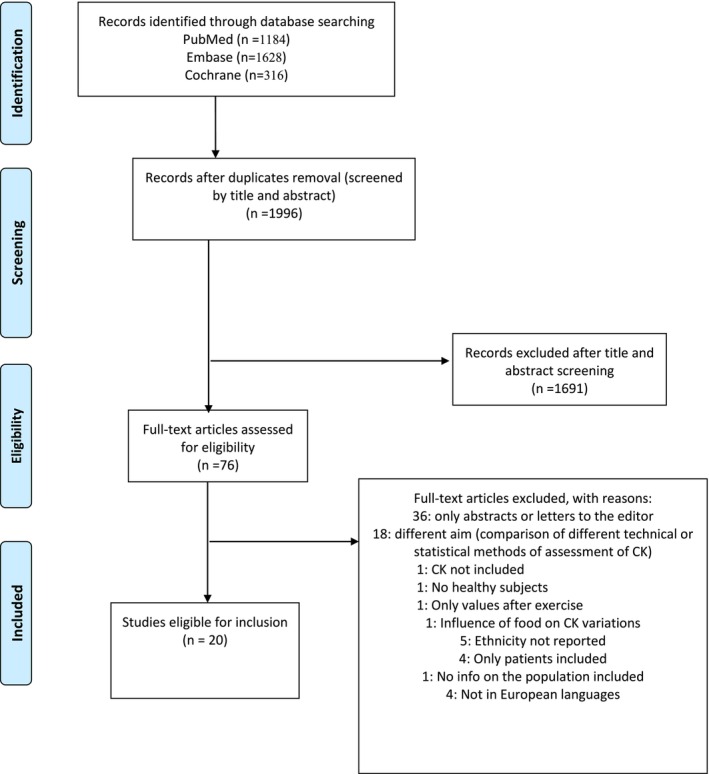
Flow diagram of the study selection process.

The literature search results are summarized in a PRISMA flowchart in Figure 1. After removing duplicates, we screened for eligibility, which included 1996 titles and abstracts and 76 full texts. The main reasons for exclusion were as follows: only abstracts were available (primarily conference abstracts), differing aims, including laboratory manuals or statistical methods for measuring CK, opinion papers, or insufficient information on the population studied or measurements inconsistent with our scope (children only, non‐healthy participants, assessment of CK after exercise). Ultimately, 20 papers were considered eligible for inclusion in our review. No additional studies were identified from the references of the included documents or the updated search conducted after finalizing the manuscript.

### 3.2 Characteristics of Included Studies

Table S1, reports the characteristics of the included studies. Table S2 divides the reference values reported in each study into various groups. We also report the methods used to obtain the reference values, such as treating outliers and applying statistical approaches (Table S2).

Of the eligible studies, eight included Caucasian populations, and the number of participants varied between 236 and 12,828 [2, 4, 12, 13, 14, 15, 16, 17]. Black populations were included in six studies, and the number of participants varied between 570 and 2101 [4, 13, 17, 18, 19, 20] Asian populations were involved in eight studies [4, 13, 17, 21, 22, 23, 24, 25], with 213 to 8973 participants per study. In two studies, Hispanic populations were included (563 and 556 participants, respectively) [13, 17]; in two studies, Turkish populations [26, 27] and one study, 230 Arab individuals were included [28]. Seven studies were from European countries [2, 4, 12, 14, 16, 26, 27]; two studies were from the USA [13, 17]; five studies were from Asia (China, Japan, and India) [21, 22, 23, 24, 25]; two studies were from Middle Eastern countries (Saudi Arabia and Israel) [15, 28] and three studies from Africa (Kenya, Ghana, and various countries from eastern and south‐eastern Africa) [18, 19, 20]. All studies reported the results for males and females separately. Most of them used similar definitions of a healthy population in their inclusion criteria, controlled for physical activity, and standardized methods for measuring CK. The reference values were calculated non‐parametrically, reporting the 2.5 and 97.5 percentiles, according to the IFCC recommendations [29, 30, 31, 32] by eight studies [2, 4, 13, 16, 17, 18, 22, 27]; six studies applied bootstrap methods [12, 14, 15, 23, 24, 33], and six studies used log transformation before calculating the percentiles [19, 20, 21, 25, 26, 28]. Correction for outliers was done using the Dixon method, also recommended by the IFCC, in six studies [4, 13, 18, 22, 25, 27]; the LAVE method in six studies [20, 21, 23, 24, 28, 33]; two studies used the Tukey or other techniques [16, 26]; it was not controlled in one study [19] and it was not reported in detail in five studies [2, 12, 14, 15, 17] (Table S2). Most of the studies included participants aged between 20 and 65; one included only a population between 20 and 25 [15], and six studies included older individuals [2, 12, 14, 21, 22, 26, 33]. In studies where CK values differed significantly by age groups, different reference values were proposed, respectively, but this division was not homogenous among studies to provide a meaningful universal comparison (Table S1).

### 3.3 Risk of Bias Assessment

The risk of bias (ROB) assessment results for each group are presented in Table S3. Most of the studies included participants who represented the general healthy population, apart from one that included army service personnel [15] and three that did not provide any description [24, 25, 27]. All studies provided a clear description of the inclusion and exclusion criteria. Still, some did not provide a sufficient description of the source of the population that was finally included or the reasons for excluding potential participants [16, 17, 21, 24, 33]. Apart from three studies that did not provide information on whether they controlled the validity of the laboratory measures [12, 14, 17], most studies used standard measurement and control methods. Seven studies did not control for physical activity [15, 16, 17, 18, 19, 22, 27] or did not use any techniques to address this confounding factor. Five studies either did not control for outliers or did not sufficiently describe their methods [12, 14, 15, 17, 26], which is a major issue that may introduce bias during the measurement of the outcome stage (reference CK level) and the statistical analysis stage if not controlled. In addition, two studies did not report whether the data were successfully normalized after log transformation, which makes the appropriateness of the statistical analysis unclear [25, 26]. Finally, ten studies did not present the median or mean values of CK in their population, which led to the exclusion of these studies in the meta‐analysis [2, 13, 16, 19, 20, 21, 24, 25, 27, 28].

### 3.4 Summary of the Reference Values Proposed in the Studies

The reference values reported in each study, categorized by race and sex, are shown in (Tables S1 and S2). Tables 1 and 2 below summarizes the reported reference interval ranges, classified by the number of participants included in the studies.

**TABLE 1:** Summary reference values for males.

CK ranges in studies with < 400 participants	CK ranges in studies with > 400 participants	Pooled median (n of studies)	Median as reported (if one study in a group)	Pooled lower values CI	Pooled upper values CI
**Turkish**					
22–42 to 206–228	48 to 227		97		
**Asian**					
47 to 641 South Asian;	43.2–69 to 230–341	120.57 (3)		100.87	140.27
**Caucasian**					
30–59 to 225–282	14–60 to 280–440	114.76 (3)		91.04	138.48
**Black**					
72–93 to 460–786	52–71 to 520–801	206.21 (3)		153.4	259.03
**Hispanic**					
572 (UL)			115		
**Arabic**					
54–266					

**TABLE 2:** Summary of reference values in females.

CK ranges in studies with < 400 participants	CK ranges in studies with > 400 participants	Pooled median (n of studies)	Median as reported (if one study in a group)	Pooled lower values CI	Pooled upper values CI
**Turkish**					
18–31 to 142–184	32 to 135		60		
**Asian**					
27 Chinese, 37 South Asian to 127 and 313	37–44 to 153–192	70.6 (3)		59.5	81.7
**Caucasian**					
18–29 to 144–201	35–50 to 200–248	NA			
**Black**					
35–58 to 290–476	49–354	143.5(2)		102.5	182.5
**Hispanic**					
29 to 145–279			65		
**Arabic**					
27–138					

### 3.5 Comparison Between Different Racial Groups and Pooled Estimates of Medians in Different Sex and Racial Groups

**FIGURE 2:**
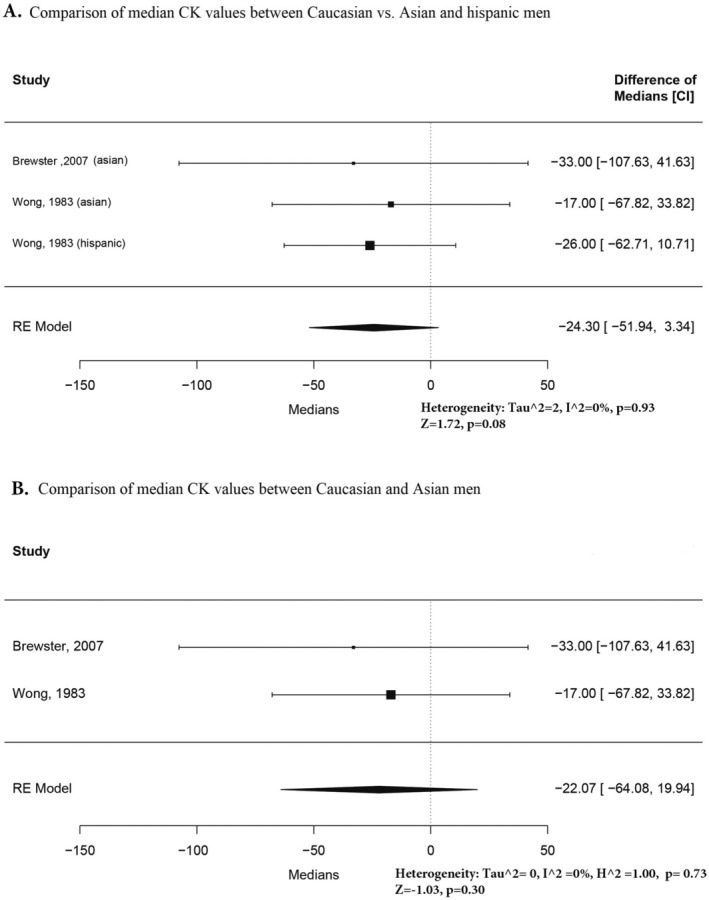
(A) Comparison of median CK values between Caucasian vs. Asian, and Hispanic men. (B) Comparison of median CK values between Caucasian and Asian men.

**FIGURE 3:**
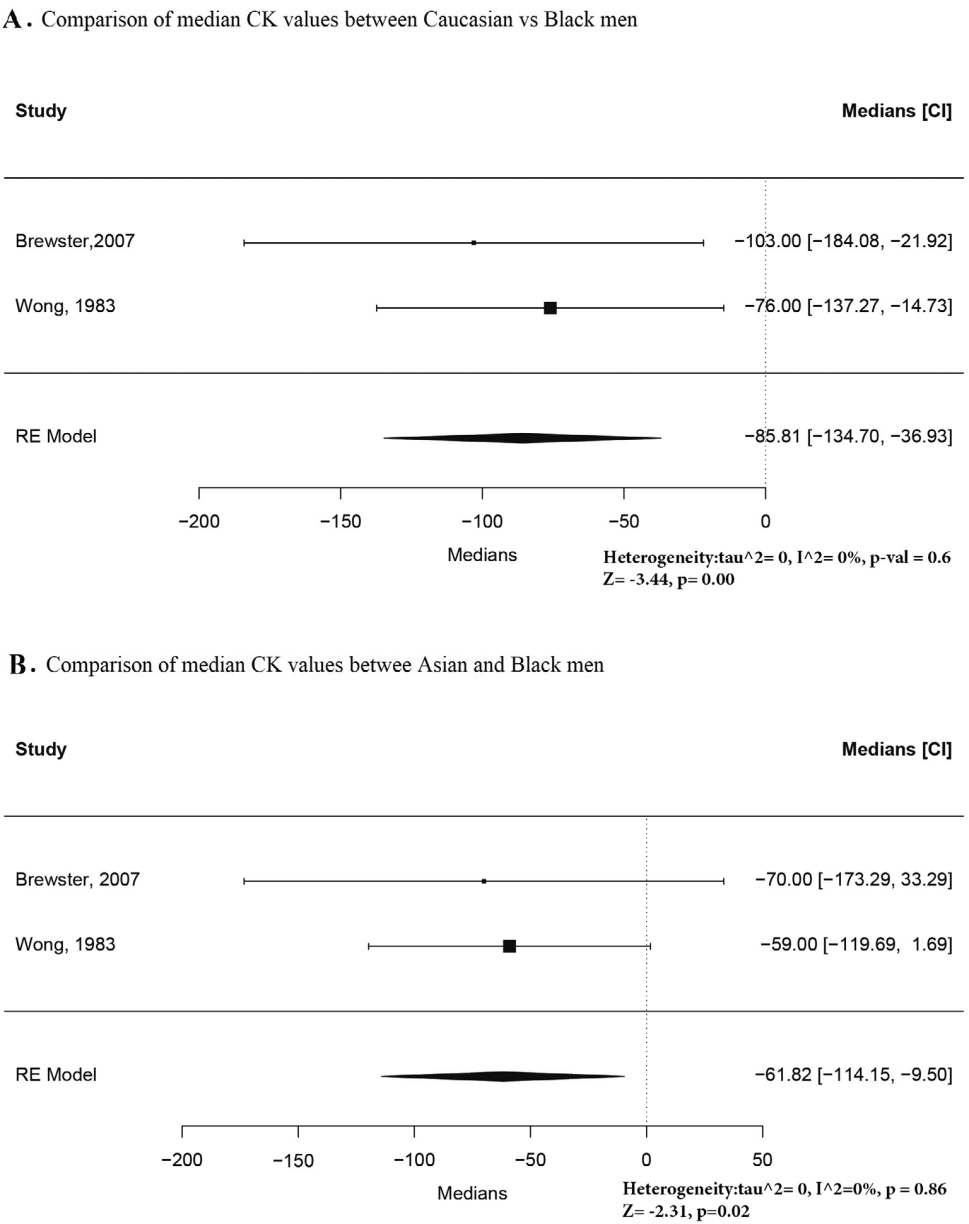
(A) Comparison of median CK values between Caucasian vs. black men. (B) Comparison of median values between Asian and Black men.

**FIGURE 4:**
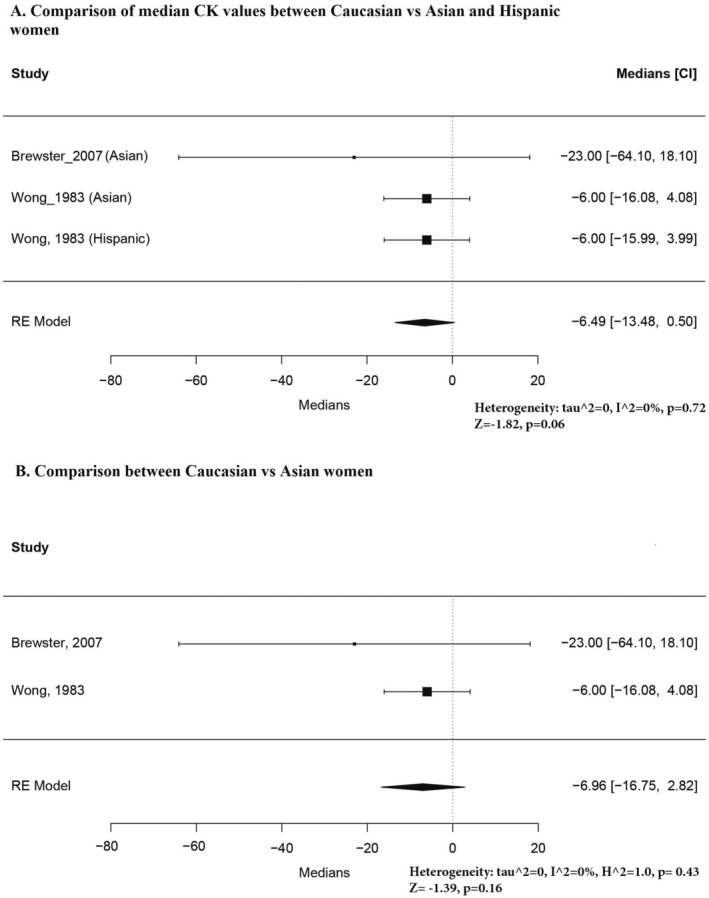
(A) Comparison of median CK values between Caucasian vs. Asian, and Hispanic women. (B) Comparison between Caucasian and Asian women.

**FIGURE 5:**
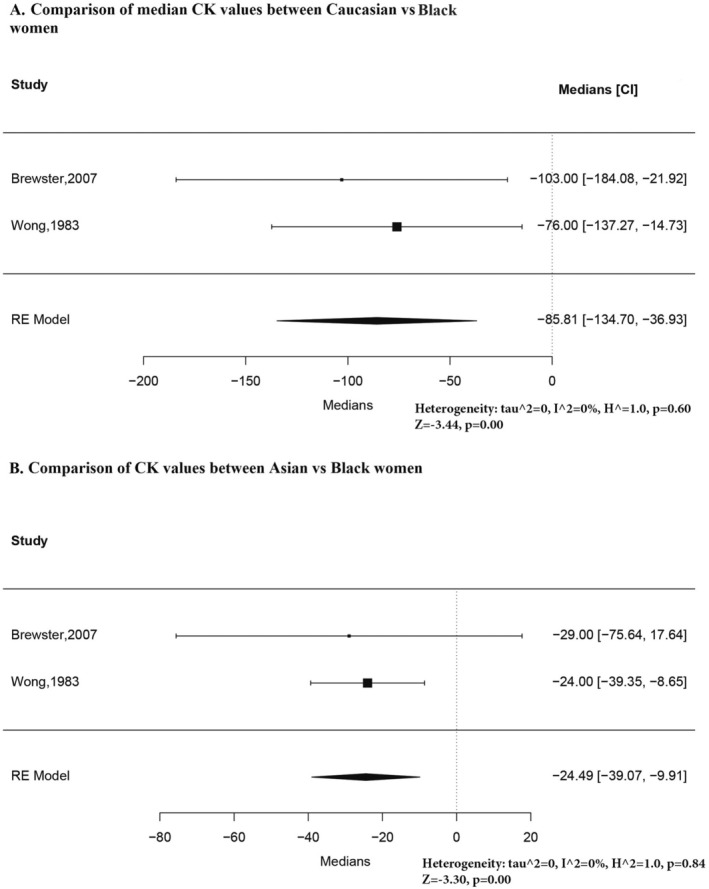
(A) Comparison of median CK values between Caucasian vs. Black women. (B) Comparison of CK values between Asian and Black women.

We included only the studies with more than one racial group (Figures 2, 3, 4, 5) to compare the reference values between groups.

Figure 2A presents the comparison between Caucasian and Asian/Hispanic males. The reason for including Asians and Hispanics is that the primary studies did not report any statistically significant differences [4, 17]. Our analysis confirmed this, showing no significant difference between the reference values for Caucasians and the Asian/Hispanic group (*I* ^2^ = 0%, *p* = 0.93). However, when we performed a pooled analysis for all non‐black males to estimate a standard median value of CK, there was a significant heterogeneity (Figure S5) resolved only when grouping by racial groups (Figures S1 and S2). For this reason, we excluded Hispanics from the comparison as the least prevalent group, and the differences between Caucasian and Asian males remain insignificant (Figure 2B).

Two studies were eligible to compare the median difference between the CK reference intervals of Caucasian and Black males and Asian and Black males (Figures 3A,B). This analysis showed significant differences in both groups.

The results of the pooled medians for each male racial group are presented in Figures S1 and S2. For this analysis, we included the studies that reported the medians or means for any individual racial group. For Caucasians, the heterogeneity was only partially resolved, so we excluded one outline with very few participants [26]. The pooled median values and lower and upper confidence interval limits for different racial groups in males are also presented in Table 1.

Figure 4A compares Caucasian and Asian/Hispanic females, showing no significant differences. Following the same approach, we excluded the Hispanic population study (Figure 4B). Once again, high heterogeneity was apparent when we attempted to estimate a standard median value (Figure S6). This issue could not be resolved by removing outliers or studies with poor ROB assessments, necessitating a division by racial groups.

Figure 5A,B compares Caucasian and Black females and Asian and Black females. For males, the difference was much more pronounced between Caucasians and Blacks, but the median differences were statistically significant in both cases.

The results of the pooled medians for each racial group among females are presented in Figures S3 and S4. A high level of heterogeneity was observed for Caucasian females; the sensitivity analysis is presented in Figure S7. The high heterogeneity was only partially resolved after removing one of the outliers with much larger values than the rest [2]. The results of this analysis should not be considered a valid estimate of the median CK values in Caucasian females. When estimating the pooled median values for Black females, heterogeneity was observed, and this was resolved upon removing one study that reported the lowest reference intervals among all included studies for Black women in this review (Figure S8) [17].

## 4 Discussion

This systematic review seeks to summarize the evidence from available studies on the normal reference values of CK across different racial groups. To our knowledge, this is the first systematic review addressing this issue.

Our results align with the findings of the individual studies included in this review [4, 17] and demonstrate that Black individuals exhibit significantly higher CK values compared to Caucasians and Asians. A similar trend is observed in both males and females. The upper reference limits in the studies included, which had more than 400 participants for Caucasian, Asian, and Turkish males, ranged from 227 to 440 U/L, while for Black males, they ranged from 520 to 810 U/L. The upper reference limits for females ranged from 135 to 248 U/L and up to 354 U/L, respectively (Tables 1 and 2). The CK values tend to exhibit a non‐Gaussian distribution, which poses challenges when synthesizing results. However, thanks to advances in statistical methods, we conducted comparative and one‐group meta‐analyses, utilizing studies that reported the ranges and the median or mean values of CK. These results indicate the need to establish new reference values for CK rather than follow traditional manufacturer reference values based not only on gender but also on racial differences. In our review, we could only descriptively summarize the reference ranges due to the data's non‐Gaussian distribution and inconsistent reporting of confidence intervals of first and third quantiles.

In general, most studies did not report the median or mean CK values and were thus excluded from the quantitative analysis. Although we could compare and estimate values for Asian and Black populations based on the eligible studies, those with the most significant number of participants primarily focused on Caucasian populations. Additional data are needed to facilitate comparisons and quantitative summaries for Hispanic and Arabic populations. Consequently, the lack of reported central values and the inconsistency in evidence availability across different racial groups may have introduced some bias into our results.

Additionally, only two studies, one involving Caucasians and another involving Turkish individuals, reported CK values for individuals over 65. The values ranged from 18 to 184 U/L for females and 22 to 206 U/L for the male Turkish population, 45 to 103 U/L for females and 59 to 125 U/L for the male Caucasian population. Since the values for this age group tend to be lower, the findings of the current meta‐analyses apply only to adults aged 18 to 65.

The definition of racial groups in the studies needed to be more clearly defined. For example, it was not clear if the Asian population included the mongoloid population only or also involved other races from South‐East Asia. Consequently, our study considered any Asian origin as one group. Also, it is unclear if the Hispanic group contains people of Native American or Latin (South European) origin. Furthermore, it was unclear if the people of Turkish origin differed from those of Arab origin. Due to the difference in study reporting, we could not correct this bias by performing a comparative quantitative analysis (e.g., between Chinese and Indian or Turkish and Arab populations). However, we tried to account for heterogeneity when we performed the pooled analysis to estimate a common median for each group (Supporting Information). Table S2 reports the values of each group as reported in each study. Future studies should report racial definitions transparently to help further distinguish the characteristics of various racial groups. Another limitation of our study is that we could not establish upper and lower limits for the reference values. Although these methods exist, they rely on normal distribution samples or necessitate individual patient data, so only descriptive analysis was feasible [34].

Future studies should report median and mean values and reference ranges for more precise estimates of group differences. Particular attention should be given to including various racial groups and older populations in cohort studies. Studies should also consider physical activity as a confounding factor in their analysis, which can introduce bias, especially if outliers are not controlled for. In our pooled analyses, all the eligible studies controlled for outliers.

Aside from these weaknesses, most studies employed a standardized method for measuring CK that was controlled for quality and clearly described the included population. Additionally, the majority of studies utilized standard estimation methods for reference values.

The main strength of our review lies in the rigorous methodology used to conduct the systematic review, ensuring that all relevant studies are included and that the current evidence is presented transparently. Moreover, while normal CK values generally exhibit a non‐Gaussian distribution in most cases, we successfully provided estimates of the differences among the three racial groups and the median values for each group separately.

In conclusion, our results, derived from a substantial and rigorous selection of papers in the CKemia field, indicate that the normal CK values might be higher than originally suggested, particularly among certain racial groups. However, future studies should standardize reporting and include diverse populations before proposing changes to the established reference values to enhance precision.

## Author Contributions

**Katina Aleksovska:** conceptualization, methodology, investigation, data curation, visualization, writing – original draft, writing – review and editing. **Theodoros Kyriakides:** conceptualization, methodology, investigation, data curation, visualization, writing – original draft, writing – review and editing. **Antonio Toscano:** conceptualization, methodology, investigation, data curation, visualization, writing – original draft, writing – review and editing. **Benedikt Schoser:** conceptualization, methodology, investigation, data curation, visualization, writing – original draft, writing – review and editing. **Corrado Angelini:** investigation, data curation, writing – review and editing. **Zohar Argov:** investigation, data curation, writing – review and editing. **Kristl G. Claeys:** investigation, data curation, writing – review and editing. **Marianne de Visser:** investigation, data curation, writing – review and editing. **Massimiliano Filosto:** investigation, data curation, writing – review and editing. **Ivanka Jovanovic:** investigation, data curation, writing – review and editing. **Anna Kostera‐Pruszczyk:** investigation, data curation, writing – review and editing. **Maria Julia Molnar:** investigation, data curation, writing – review and editing. **Sabrina Sacconi:** investigation, data curation, writing – review and editing. **Jochen Schaefer:** investigation, data curation, writing – review and editing. **Gabriele Siciliano:** investigation, data curation, writing – review and editing. **Juan J. Vilchez:** investigation, data curation, writing – review and editing.

## Conflicts of Interest

The authors declare no conflicts of interest.

## Supporting information




Figure S1.

Tables S1.

## Data Availability

Additional data is available in Appendices 1 and Supporting Information files. If not reported, please send a request to katinaaleksovska@gmail.com.

## Appendix 1 Search Terms

**Table:**

Database: PubMed **Date: May 2024**	**Search terms**	**Limits**	**Number of records**
#1	CK[Title/Abstract] OR “Creatine kinase” OR “creatine kinase”[MeSH Terms] OR creatine kinase[Text Word]		
#2	(reference OR normal OR physiologic) AND values		
#3	#1 AND #2		4803
#4	Cardiac OR myocardial OR animals		
#5	#3 NOT #4		1421

**Database: Embase** **Date: May 2024**	**Search terms**	**Limits**	**Number of records**
#1	‘creatine kinase’/exp. OR ‘creatine kinase’ OR ck:ti,ab,kw		
#2	(reference OR normal OR physiologic) AND (‘values’/exp. OR values)		
#3	#1 AND #2		3404
#4	NOT (cardiac:ti,ab,kw OR myocardial:ti,ab,kw OR animals:ti,ab,kw)		
#5	#3 NOT #4		2041

**Database: Cochrane** **Date: May 2024**	**Search terms**	**Limits**	**Number of records**
#1	CK OR Creatine kinase in Title, Abstract, Keyword		
#2	(reference OR normal OR physiologic) AND values in Title, Abstract, Keyword		
#3	#1 AND #2		553
#4	Cardiac OR myocardial OR animals in Title, Abstract, Keyword		
#5	#3 NOT #4		316
