# Supplementary material for: What Are the Normal Serum Creatine Kinase Values for Skeletal Muscle? A Worldwide Systematic Review
Source: Eur J Neurol. 2025 Jun 13;32(6):e70240. doi: 10.1111/ene.70240 (PMC12163646; doi:10.1111/ene.70240)

**Supplementary Figure 8.** Quantitative analysis of median CK values for Black females (sensitivity analysis)


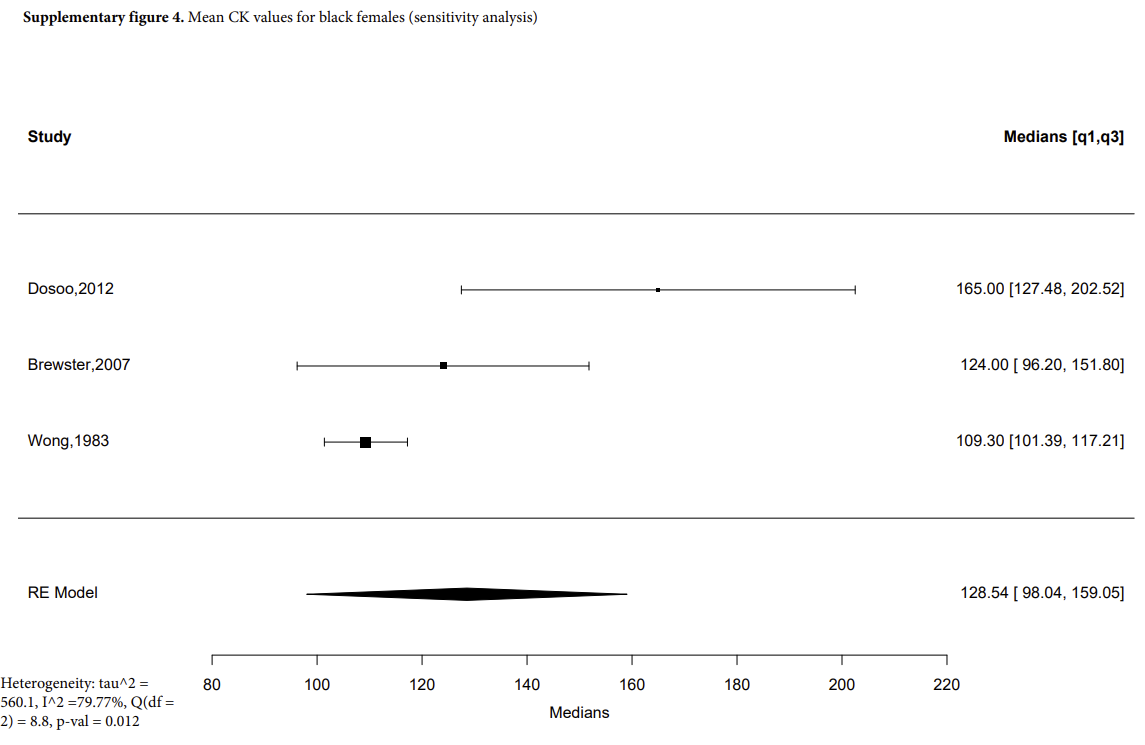

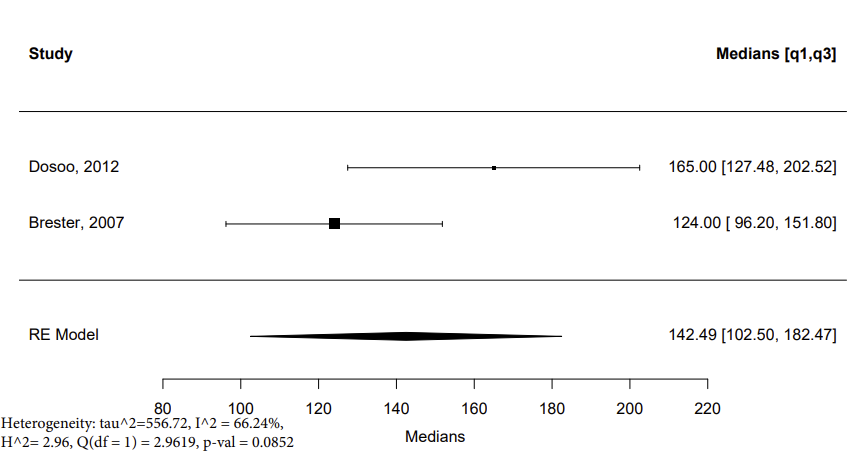

Supplement: Supplementary file 1 — Figure S1. [file ENE-32-e70240-s001.zip › Supplementary figure 8.docx]
