# Supplementary figures and images for: What Are the Normal Serum Creatine Kinase Values for Skeletal Muscle? A Worldwide Systematic Review
Source: Eur J Neurol. 2025 Jun 13;32(6):e70240. doi: 10.1111/ene.70240 (PMC12163646; doi:10.1111/ene.70240)

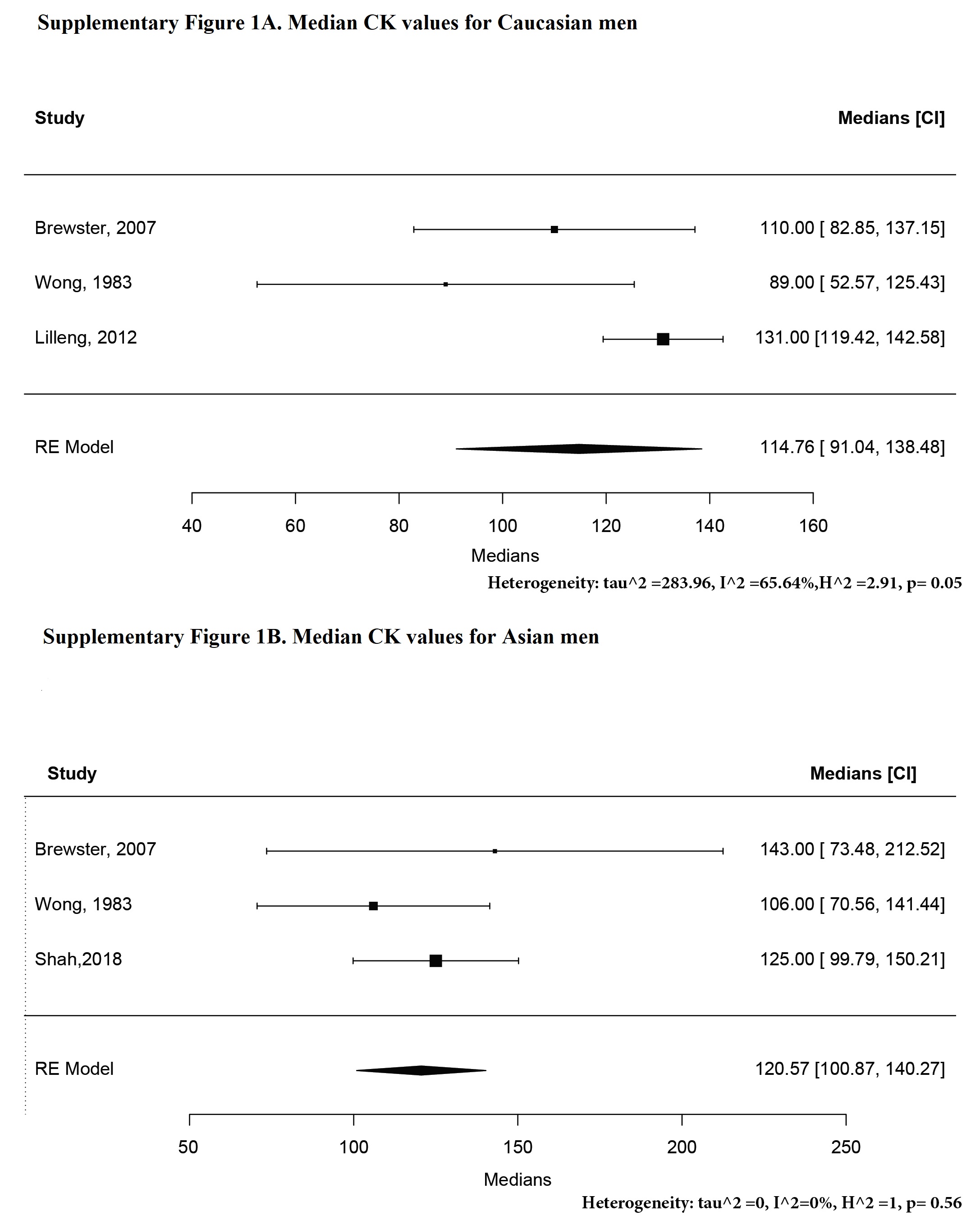

Supplement: Supplementary file 1 — Figure S1. [file ENE-32-e70240-s001.zip › Supplementary figure 1.jpg]

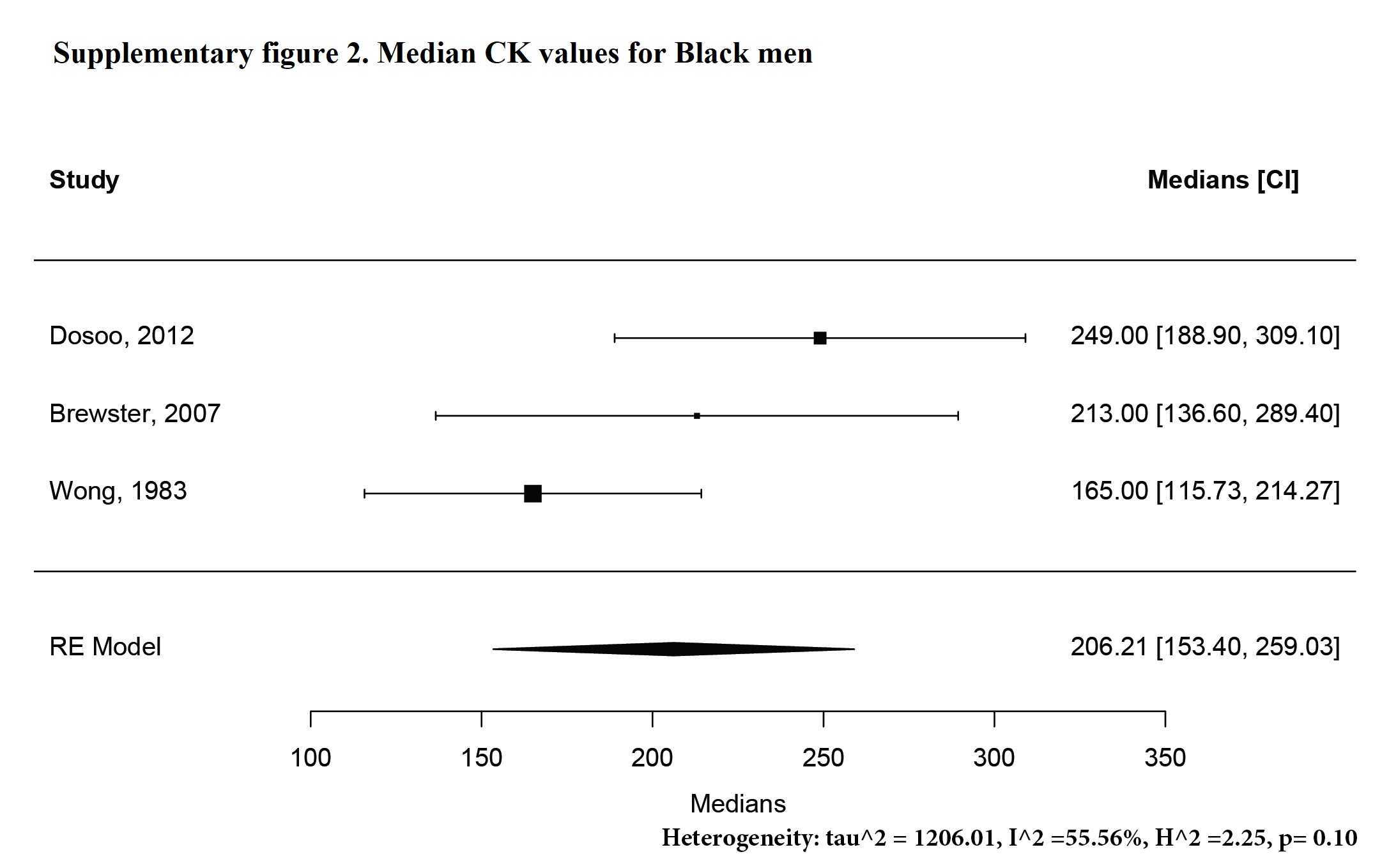

Supplement: Supplementary file 1 — Figure S1. [file ENE-32-e70240-s001.zip › Supplementary figure 2.jpg]

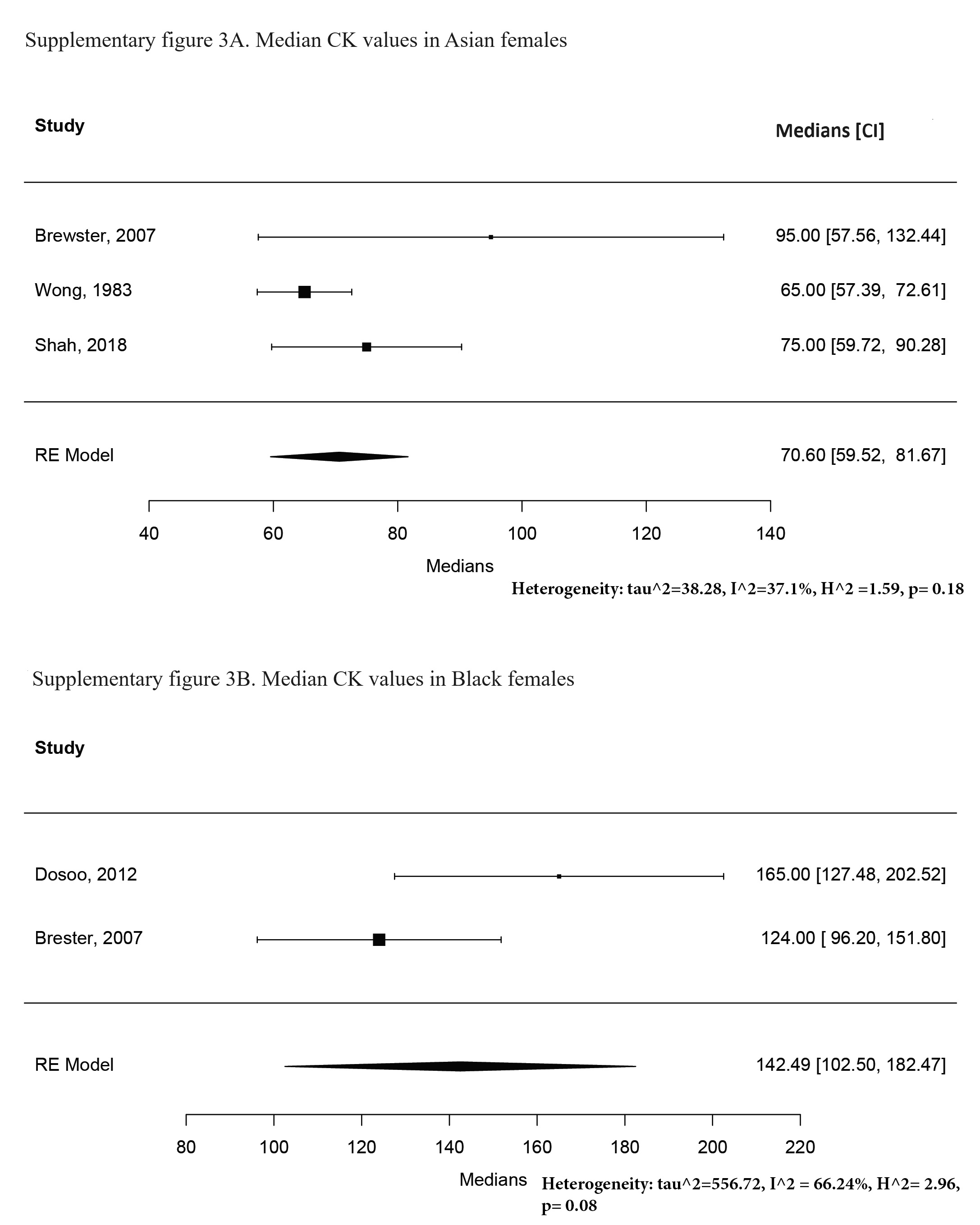

Supplement: Supplementary file 1 — Figure S1. [file ENE-32-e70240-s001.zip › Supplementary figure 3.jpg]

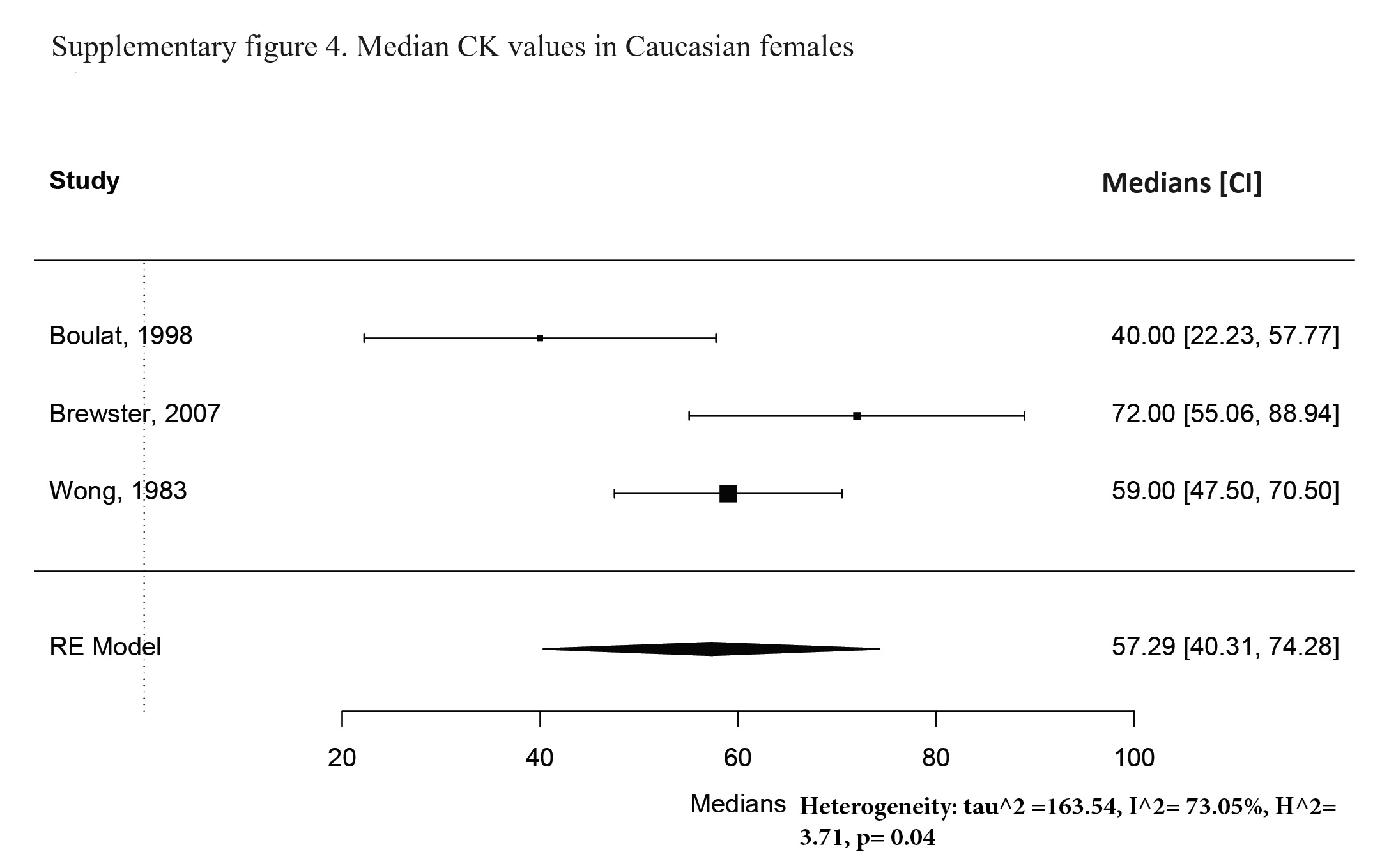

Supplement: Supplementary file 1 — Figure S1. [file ENE-32-e70240-s001.zip › Supplementary figure 4.jpg]

Supplementary figure 5. Median CK values for non-black males

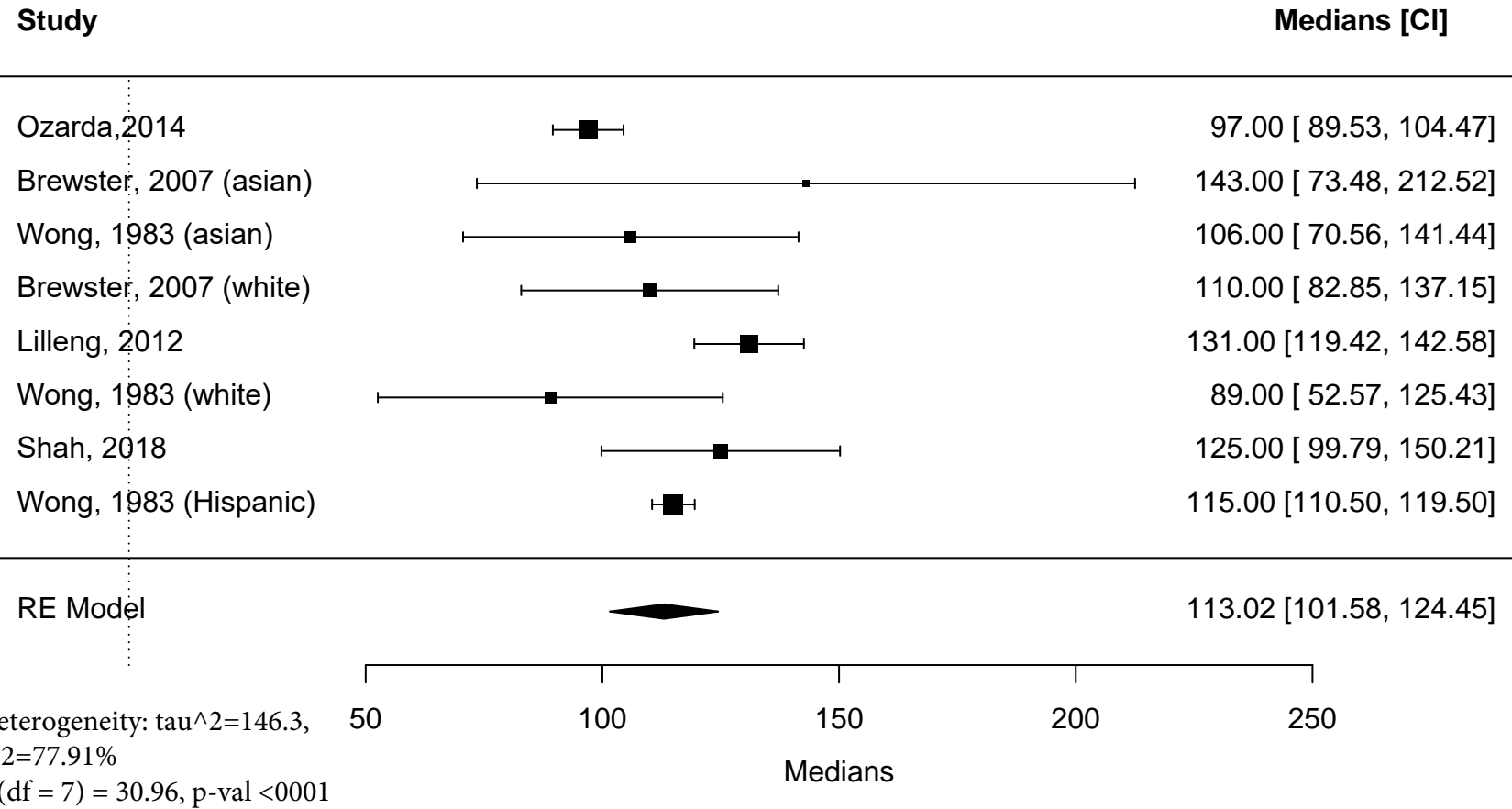

Supplement: Supplementary file 1 — Figure S1. [file ENE-32-e70240-s001.zip › Supplementary Figure 5.pdf]

Supplementary figure 6. Median CK values for non-black females

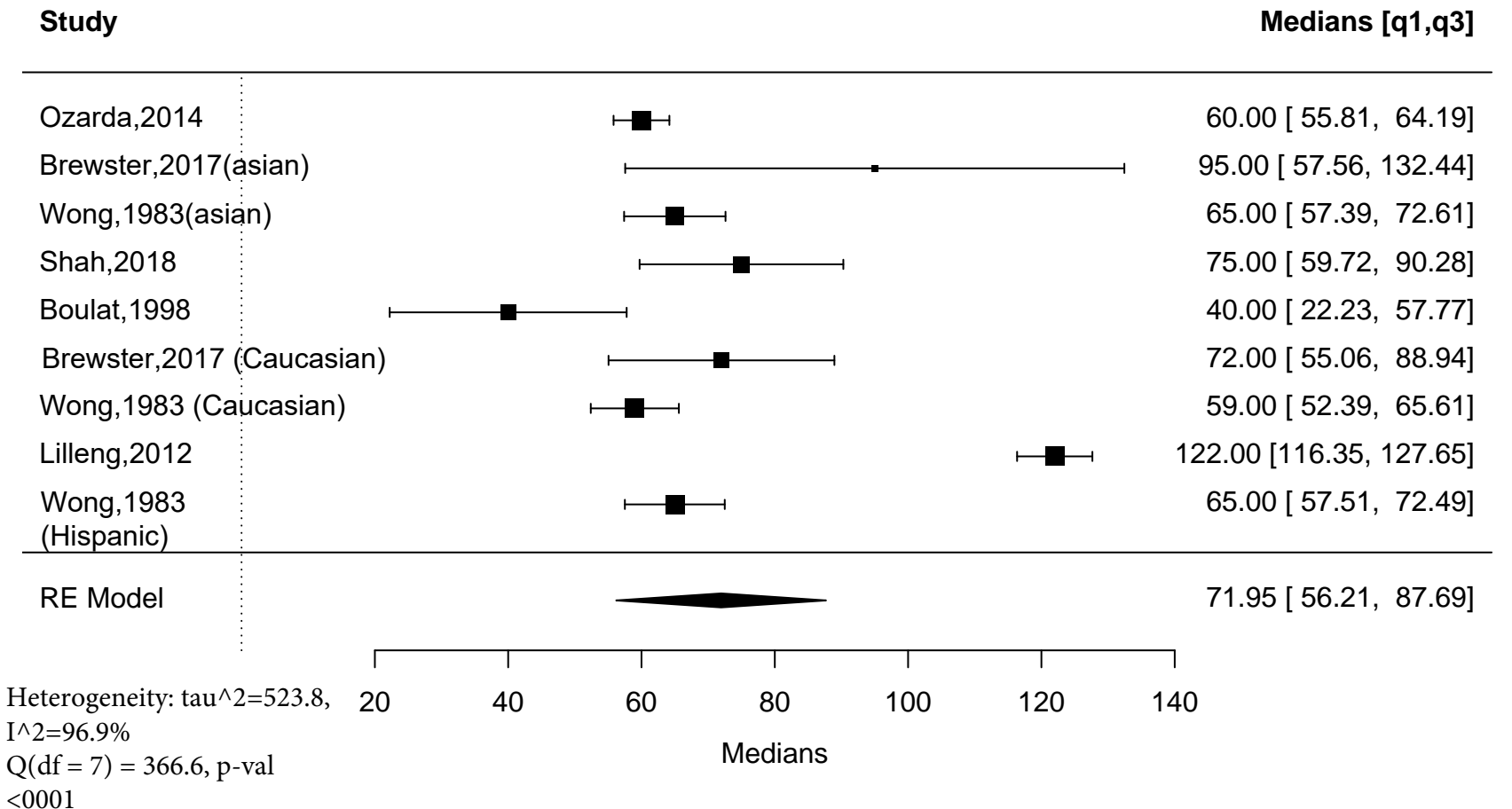

Supplement: Supplementary file 1 — Figure S1. [file ENE-32-e70240-s001.zip › Supplementary figure 6.pdf]
